# Phenylephrine does not improve oxygenation during one-lung ventilation: A randomized, double-blind, cross-over study

**DOI:** 10.1371/journal.pone.0195576

**Published:** 2018-04-09

**Authors:** Kohei Godai, Maiko Hasegawa-Moriyama, Akira Matsunaga, Yuichi Kanmura

**Affiliations:** Department of Anesthesiology and Critical Care Medicine, Graduate School of Medical and Dental Sciences, Kagoshima University, Kagoshima, Japan; Tokai Daigaku, JAPAN

## Abstract

**Background:**

Phenylephrine is an α_1_ adrenergic receptor agonist that causes pulmonary vasoconstriction, and so may effectively enhance hypoxic pulmonary vasoconstriction (HPV). However, there is little evidence that phenylephrine augments HPV in clinical situations. This study aimed to evaluate the clinical effects of phenylephrine infusion on oxygenation during one-lung ventilation (OLV) in patients undergoing thoracic surgery.

**Methods:**

This was a prospective, randomized, double-blind, cross-over study. Included patients were those undergoing elective thoracic surgery in the lateral decubitus position with OLV. Patients were randomly allocated to two groups. The N-P group initially had OLV with normal saline infusion for 30 minutes; after a 10 minute interval, OLV was then maintained with phenylephrine infusion for 30 minutes. The P-N group had the drug-infusion in the reverse order. The primary outcome was arterial partial pressure of oxygen. Secondary outcomes were mean arterial pressure, heart rate, pulse pressure variation, perfusion index, and difference between bladder and skin temperature. Statistical analysis was performed using the student t-test, Fisher's exact test, and ANOVA for Cross-over design. *P* < 0.05 was considered statistically significant.

**Results:**

Twenty-nine patients were analyzed. Although phenylephrine infusion significantly increased mean arterial pressure (*P* < 0.001), arterial partial pressure of oxygen did not differ between the two timepoints (*P* = 0.19). There was no carryover effect in arterial partial pressure of oxygen (*P* = 0.14). Phenylephrine infusion significantly decreased heart rate (*P* = 0.02) and pulse pressure variation (*P* < 0.001).

**Conclusions:**

Phenylephrine infusion did not improve oxygenation during OLV. The present results indicate that phenylephrine does not have clinically meaningful effects on HPV.

**Trial registration:**

University Hospital Medical Information Network 000024317

## Introduction

Hypoxemia during one-lung ventilation (OLV) occurs in 5–10% of patients, and may affect the patient safety[1]. Hypoxemia is primarily caused by the shunt through the non-dependent lung, with the degree of venous mixture mainly determined by the distribution of perfusion[2]. Perfusion of the non-dependent lung is reduced by multiple factors, one of which is hypoxic pulmonary vasoconstriction (HPV). HPV is a reflex contraction of vascular smooth muscle in the lung in response to regional hypoxia[3], which contributes to maintaining oxygenation during OLV. HPV is attenuated by intravenous vasodilators such as prostacyclin or calcium antagonists, and thus these drugs worsen the oxygenation status.

Phenylephrine is an α_1_ adrenergic receptor agonist that causes pulmonary vasoconstriction. Phenylephrine administration may effectively enhance HPV[3]; however, there is little evidence that phenylephrine augments HPV in clinical situations. Two case reports showed that phenylephrine infusion improved oxygenation during general anesthesia[4, 5]. To the best of our knowledge, only one clinical study has prospectively evaluated phenylephrine infusion as a therapy for augmenting HPV in hypoxemic patients[6], reporting that phenylephrine improved oxygenation in patients with acute respiratory distress syndrome[6]. Our hypothesis was that phenylephrine infusion increased arterial partial pressure of oxygen (PaO_2_) during OLV. The aim of the present study was to evaluate the clinical effects of phenylephrine infusion on oxygenation during OLV in patients undergoing thoracic surgery.

## Materials and methods

This manuscript adheres to the applicable CONSORT guidelines. The supporting CONSORT checklist and protocol are available as S1 Fig, S1 File, S2 File. This prospective, randomized, double-blind, cross-over study was approved by the ethics committee of Kagoshima University Hospital, and was conducted from October 2016 to November 2017 at Kagoshima University Hospital. This trial was prospectively registered on a publicly accessible database (UMIN Clinical Trials Registry ID: UMIN000024317). Written informed consent was obtained from all participants.

Inclusion criteria were: patients with American Society of Anesthesiologists physical status I–III undergoing elective thoracic surgery in the lateral decubitus position with at least 70 minutes of OLV. The original exclusion criteria were: history of stroke, uncompensated cardiac disease, or bradycardic arrhythmia. However, as arrhythmia affects the measurement of pulse pressure variation (PPV)[7], we decided to exclude all arrhythmic cases after the completion of the third case. None of the patients treated before the exclusion criteria were updated had arrhythmia. We changed the database information on 24th August 2017.

### Protocol

Before induction of general anesthesia, a thoracic epidural catheter (17G Tuohy needle, Hakko disposable epidural catheter; Hakko, Japan) was placed at T4 to T7 according to the incision level, and 3 mL mepivacaine 1% without epinephrine was administered. General anesthesia was induced with target-controlled infusion of propofol (2–4 μg/mL), remifentanil (0.3 μg/kg/min), and rocuronium (0.6 mg/kg). Anesthesia was maintained with target-controlled infusion of propofol (2–4 μg/mL) and remifentanil (0.1–0.5 μg/kg/min), and intermittent bolus administration of rocuronium (10 mg). Bispectral index (BIS) values were monitored using a BIS Quatro sensor (Covidien, Mansfield, MA, USA), and the propofol infusion rate was adjusted to maintain BIS values between 40 and 60. No volatile anesthetics were used. The rate of remifentanil infusion was tailored to control hemodynamic responses. Rocuronium was used to maintain a train-of-four ratio of 1 or less (TOF-Watch SX, Organon, Ireland). Heart rate (HR), direct arterial blood pressure, electrocardiogram, BIS, peripheral oxygen saturation (SpO_2_), end-tidal carbon dioxide tension (ETCO_2_), PPV, perfusion index (PI) derived from the pulse oximeter plethysmographic waveform, bladder temperature, and skin temperature of the hand were continuously monitored (Life Scope J, Nihon Kohden, Japan). Intravenous fluid therapy consisted of 6% hydroxyethyl starch in saline (Voluven, Fresenius Kabi Japan, Japan) at a rate of 80 ml/h, and 4.3% dextrose solution (Soldem 3A, Terumo, Japan) at a rate of 20 ml/h. If the mean arterial pressure (MAP) was less than 50 mmHg, a bolus of ephedrine (4 mg) was administered.

The trachea was intubated with a left-sided double-lumen tube (DLT) (Blue Line, Smiths Medical International, UK: 35 or 37 F for males and 32 or 35 F for females). Positioning of the DLT was confirmed by fiberoptic bronchoscopy before and after placing the patient in the lateral decubitus position. Lung separation was confirmed by auscultation. During OLV, the lumen of the non-dependent lung was left open to air. The patients’ lungs were ventilated using an Aisys Pro anesthetic machine (GE Healthcare, Chicago, IL, USA) with an inspired oxygen fraction (FIO_2_) of 1.0, tidal volume of 5–7 mL/kg of ideal bodyweight, and respiratory rate of 12 breaths/min; this was adjusted to maintain an ETCO_2_ of 35–45 mm Hg and a positive end-expiratory pressure of 6 cmH_2_O. The patients were randomly allocated to one of two groups using internet-based software in a complete randomization manner (Research Randomizer version 4.0, retrieved on October 13, 2016 from http://www.randomizer.org/). The allocation was blinded for the patients, anesthesiologists, and surgeons. In the N-P group, 10 minutes after each patient’s chest was opened, OLV was initially maintained with a normal saline infusion (20 mL/h) for 30 minutes; after a 10 minute interval, OLV was then maintained with a phenylephrine infusion (15 μg/min) for 30 minutes. In the P-N group, 10 minutes after each patient’s chest was opened, OLV was initially maintained with a phenylephrine infusion (15 μg/min) for 30 minutes; after a 10 minute interval, OLV was then maintained with a normal saline infusion (20 mL/h) for 30 minutes. At the end of each drug infusion, arterial blood analysis was performed with a blood gas analyzer (ABL700, Radiometer, Denmark), and the MAP, HR, PPV, and PI were recorded. These measurements were recorded before any major pulmonary vessel clipping. The anesthetic management after these measurements was at the discretion of the anesthesia care provider.

### Data analysis

The primary outcome was PaO_2_. The secondary outcomes were MAP, HR, PPV, PI, and the difference between the bladder and skin temperature (ΔT). Originally, we calculated the required sample size as 35 patients using between-subject variation as follows. To detect a difference of 30 mmHg in PaO_2_ during OLV with a two-sided approximation accepting an α error of 5% and a β error of 20%, the required study size was calculated as 29 patients based on a previous study using Power and Sample Size Calculation version 3.1.2 (Dupont WD and Plummer WD, Vanderbilt University, Nashville, TN)[8]. To account for patients dropping out during the study, 20% more patients were added, giving a final sample size of 35 patients. However, after completion of the study, we recognized that the sample size estimation should be based on within-individual variation, not on between-subject variation[9]. Hence, we performed a power analysis. The standard deviation of within-individual variation in PaO_2_ during OLV in 40 minutes was 33 mmHg in the present study. To detect a difference of 30 mmHg in PaO_2_ with a two-sided approximation accepting an α error of 5%, the power was calculated as 0.925. All data were expressed as means with standard deviations or with 95% confidence intervals. Statistical analysis was performed using the student *t*-test, Fisher's exact test, and ANOVA for Cross-over design (GraphPad Prism 5.0, La Jolla, CA, USA). *P* < 0.05 was considered statistically significant. All data were deposited in the public repository (https://upload.umin.ac.jp/cgi-open-bin/ctr/ctr_view.cgi?recptno=R000028009).

## Results

**Fig 1:**
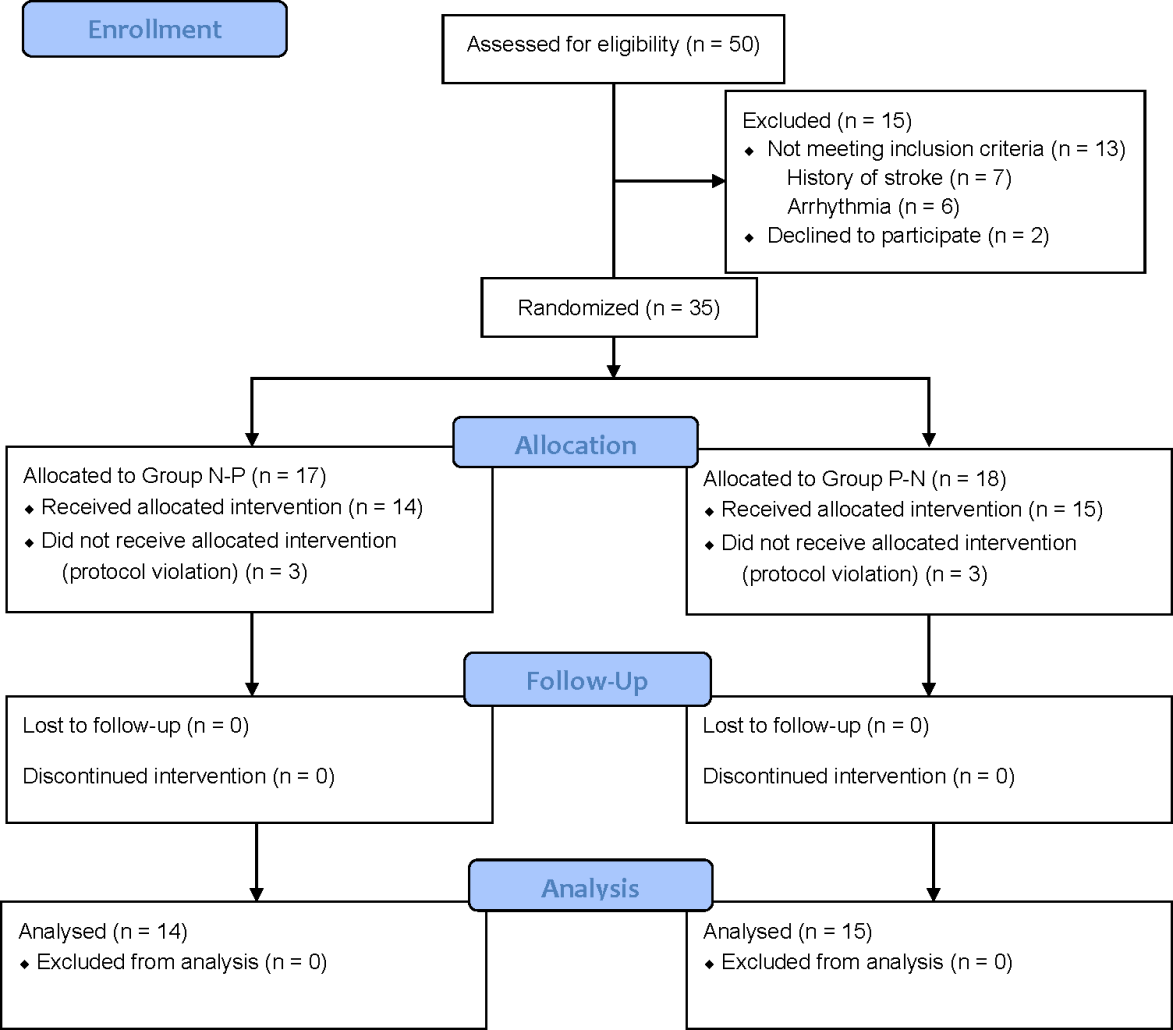
The CONSORT flow diagram of the study.

The CONSORT diagram is shown in Fig 1.

Of 50 patients considered eligible for the study, 13 patients were excluded due to a history of stroke or presence of a current arrhythmia, and two patients declined to participate. A total of 35 patients were included in the study; however, only the data from 29 patients were analyzed, as six patients did not receive their allocated intervention due to protocol violations. Table 1 shows the patients’ and surgical characteristics.

**Table 1:** Demographic, preoperative, and intraoperative clinical data.

	Group N-P (n = 14)	Group P-N (n = 15)	*P* value
Age (y)	68 ± 11	68 ± 9	0.95^a^
Male Sex (n)	5	3	0.43^b^
Height (cm)	161 ± 9	154 ± 9	0.06^a^
Weight (kg)	59 ± 11	54 ± 14	0.29^a^
ASA Physical Status (1 / 2 / 3)	1 / 13 / 0	2 / 11 / 2	0.99^b^
Left Side of Surgery (n)	6	6	0.99^b^
Types of Surgery			0.65^b^
Pneumonectomy (n)	0	1	
Bilobectomy (n)	1	0	
Lobectomy (n)	11	13	
Segmentectomy (n)	1	0	
Wedge resection (n)	1	1	
Preoperative FVC (% predicted)	103 ± 19	104 ± 14	0.94^a^
Preoperative FEV1 (% predicted)	75 ± 11	76 ± 5	0.65^a^
Duration of Anesthesia (min)	374 ± 105	350 ± 59	0.46^a^
Duration of Surgery (min)	267 ± 86	235 ± 55	0.24^a^
Intraoperative Fluid Load (L)	1648 ± 717	1383 ± 592	0.29^a^
Intraoperative Urine Output (L)	408 ± 262	557 ± 431	0.27^a^
Intraoperative Blood Loss (L)	80 ± 109	87 ± 191	0.91^a^
Intraoperative Fluid Balance (L)	1188 ± 555	872 ± 297	0.06^a^

Abbreviations: ASA, American Society of Anesthesiologists; FEV1, forced expiratory volume in 1 second; FVC, forced vital capacity.

Values expressed as mean ± standard deviation or number.

^a^*P* values were calculated using the student *t*-test.

^b^*P* values were calculated using the Fisher's exact test.

There was no significant difference between groups in the patient demographics and surgical characteristics. Most patients underwent lobectomy. No patient had severe respiratory dysfunction, received preoperative β-blocker medications, or was transfused perioperatively. Table 2 shows the patients’ baseline hemodynamic and blood gas analysis data taken 10 minutes after the chest was opened, immediately before the interventions. HR was significantly higher in the P-N group than in the N-P group (*P* = 0.04), although this difference was only a mean of 6 beats/min. No patient was hypoxemic before the interventions.

**Table 2:** Baseline hemodynamic and arterial blood gas data immediately before the interventions.

	Group N-P (n = 14)	Group P-N (n = 15)	*P* value^a^
PaO_2_ (mmHg)	143 ± 53	181 ± 72	0.12
MAP (mmHg)	75 ± 11	79 ± 11	0.26
HR (beats/min)	70 ± 7	76 ± 7	0.04
PPV (%)	7.4 ± 4.2	5.0 ± 3.0	0.09
PI (%)	1.9 ± 0.9	1.8 ± 0.8	0.77
ΔT (°C)	1.2 ± 0.6	1.5 ± 0.8	0.35
TV (mL)	338 ± 64	338 ± 84	0.98
Ppeak (cmH2O)	21 ± 3	22 ± 3	0.36
ETCO_2_ (mmHg)	38 ± 3	39 ± 3	0.69
SpO_2_ (%)	98 ± 2	99 ± 1	0.09
pH	7.35 ± 0.04	7.36 ± 0.03	0.48
PaCO_2_ (mmHg)	47 ± 5	47 ± 4	0.76
Lactate (mmol/mL)	0.8 ± 0.2	0.8 ± 0.2	0.51

Abbreviations: ΔT, difference between bladder and skin temperature; ETCO_2_, end-tidal carbon dioxide tension; HR, heart rate; MAP, mean arterial pressure; PaCO_2_, arterial partial pressure of carbon dioxide; PaO_2_, arterial partial pressure of oxygen; PI, perfusion index; Ppeak, peak inspiratory pressure; PPV, pulse pressure variation; SpO_2_, peripheral oxygen saturation; TV, total volume.

Values are expressed as mean ± standard deviation.

^a^*P* values were calculated using the student *t*-test.

The primary and secondary outcomes are shown in Tables 3–5.

**Table 3:** Primary and secondary outcomes.

	Treatment period		
Treatment sequence	1	2	Within-individual difference: N-P
**N then P**			
PaO_2_ (mmHg), mean (SD)	152 (60)	208 (92)	-56 (82)
PaO_2_ (mmHg), *n*	14	14	14
MAP (mmHg), mean (SD)	70 (10)	99 (18)	-29 (23)
MAP (mmHg), *n*	14	14	14
HR (beats/min), mean (SD)	77 (10)	72 (10)	5.8 (5.8)
HR (beats/min), *n*	14	14	14
PPV (%), mean (SD)	8.2 (6.3)	3.9 (2.9)	4.3 (4.7)
PPV (%), *n*	14	14	14
PI (%), mean (SD)	1.9 (0.7)	2.2 (1.0)	-0.3 (0.7)
PI (%), *n*	14	14	14
ΔT (°C), mean (SD)	1.0 (0.6)	1.0 (0.8)	0.0 (0.5)
ΔT (°C), *n*	14	14	14
**P then N**			
PaO_2_ (mmHg), mean (SD)	211 (78)	231 (93)	-20 (58)
PaO_2_ (mmHg), *n*	15	15	15
MAP (mmHg), mean (SD)	89 (10)	73 (8)	-16 (9)
MAP (mmHg), *n*	15	15	15
HR (beats/min), mean (SD)	74 (7)	73 (9)	-0.6 (5.8)
HR (beats/min), *n*	15	15	15
PPV (%), mean (SD)	3.8 (2.2)	6.5 (4.8)	2.7 (3.4)
PPV (%), *n*	15	15	15
PI (%), mean (SD)	2.4 (1.1)	2.2 (1.3)	-0.2 (0.8)
PI (%), *n*	15	15	15
ΔT (°C), mean (SD)	1.3 (1.2)	1.4 (1.0)	-0.1 (0.6)
ΔT (°C), *n*	15	15	15

Abbreviations: ΔT, difference between bladder and skin temperature; HR, heart rate; MAP, mean arterial pressure; N, normal saline infusion; P, phenylephrine infusion; PaO_2_, arterial partial pressure of oxygen; PI, perfusion index; PPV, pulse pressure variation; SD, standard deviation.

Values expressed as mean (SD).

**Table 4:** Primary and secondary outcomes (analysis of treatment effect).

Treatment effect	Within-individual difference: N-P
PaO_2_ (mmHg), mean (95% CI)	18 (-9 to 45)
PaO_2_ (mmHg), *n*	29
Paired analysis	*P* = 0.19^a^
MAP (mmHg), mean (95% CI)	22 (16 to 29)
MAP (mmHg), *n*	29
Paired analysis	*P <* 0.001^a^
HR (beats/min), mean (95% CI)	-2.6 (-4.8 to -0.4)
HR (beats/min), *n*	29
Paired analysis	*P* = 0.02^a^
PPV (%), mean (95% CI)	-3.4 (-5.0 to -1.9)
PPV (%), *n*	29
Paired analysis	*P* < 0.001^a^
PI (%), mean (95% CI)	0.3 (-0.0 to 0.6)
PI (%), *n*	29
Paired analysis	*P* = 0.06^a^
ΔT (°C), mean (95% CI)	0.0 (-0.2 to 0.2)
ΔT (°C), *n*	29
Paired analysis	*P* = 0.79^a^

Abbreviations: 95% CI, 95% confidence interval; ΔT, difference between bladder and skin temperature; HR, heart rate; MAP, mean arterial pressure; PaO2, arterial partial pressure of oxygen; PI, perfusion index; PPV, pulse pressure variation.

Values expressed as mean (95% CI).

^a^*P* values were calculated using ANOVA for Cross-over design.

**Table 5:** Primary and secondary outcomes (analysis of carryover effect and period effect).

	Within-individual difference: N-P
**Carryover effect**	
PaO_2_ (mmHg), mean (95% CI)	83 (-11 to 177)
PaO_2_ (mmHg), *n*	29
Paired analysis	*P* = 0.14^a^
MAP (mmHg), mean (95% CI)	-8 (-19 to 3)
MAP (mmHg), *n*	29
Paired analysis	*P* = 0.24^a^
HR (beats/min), mean (95% CI)	-1.5 (-12.3 to -9.3)
HR (beats/min), *n*	29
Paired analysis	*P* = 0.81^a^
PPV (%), mean (95% CI)	-1.8 (-6.7 to 3.0)
PPV (%), *n*	29
Paired analysis	*P* = 0.53^a^
PI (%), mean (95% CI)	0.5 (-0.8 to 1.8)
PI (%), *n*	29
Paired analysis	*P* = 0.52^a^
ΔT (°C), mean (95% CI)	0.7 (-0.5 to 1.8)
ΔT (°C), *n*	29
Paired analysis	*P* = 0.34^a^
**Period effect**	
PaO_2_ (mmHg), mean (95% CI)	38 (11 to 65)
PaO_2_ (mmHg), *n*	29
Paired analysis	*P* = 0.007^a^
MAP (mmHg), mean (95% CI)	7 (-0 to 13)
MAP (mmHg), *n*	29
Paired analysis	*P* = 0.05^a^
HR (beats/min), mean (95% CI)	-3.2 (-5.4 to -1.0)
HR (beats/min), *n*	29
Paired analysis	*P* = 0.006^a^
PPV (%), mean (95% CI)	-0.8 (-2.4 to 0.8)
PPV (%), *n*	29
Paired analysis	*P* = 0.30^a^
PI (%), mean (95% CI)	0.0 (-0.2 to 0.3)
PI (%), *n*	29
Paired analysis	*P* = 0.73^a^
ΔT (°C), mean (95% CI)	-0.0 (-0.2 to 0.2)
ΔT (°C), *n*	29
Paired analysis	*P* = 0.67^a^

Abbreviations: 95% CI, 95% confidence interval; ΔT, difference between bladder and skin temperature; HR, heart rate; MAP, mean arterial pressure; PaO_2_, arterial partial pressure of oxygen; PI, perfusion index; PPV, pulse pressure variation.

Values expressed as mean (95% CI).

^a^*P* values were calculated using ANOVA for Cross-over design.

No patient had difficulty with operated lung isolation using the DLT during OLV. There were no hypoxemic episodes or vasodilator administrations during the study period. Although phenylephrine infusion significantly increased the MAP by about 30%, the PaO_2_ did not significantly differ between the two timepoints. Phenylephrine infusion significantly decreased HR and PPV. There was no difference in PI and ΔT between the two timepoints. There was no carryover effect in the primary and secondary outcomes. The period significantly affected the PaO_2_ and HR.

The hemodynamic and arterial blood gas data are shown in Tables 6–8.

**Table 6:** Hemodynamic and arterial blood gas data.

	Treatment period		
Treatment sequence	1	2	Within-individual difference: N-P
**N then P**			
TV (mL), mean (SD)	352 (80)	344 (79)	8 (24)
TV (mL), *n*	14	14	14
Ppeak (cmH_2_O), mean (SD)	24 (3.6)	25 (3.6)	-1.2 (1.6)
Ppeak (cmH_2_O), *n*	14	14	14
ETCO_2_ (mmHg), mean (SD)	37 (3.1)	39 (3.4)	-1.2 (2.0)
ETCO_2_ (mmHg), *n*	14	14	14
SpO_2_ (%), mean (SD)	99 (1.3)	99 (1.5)	-0.1 (0.7)
SpO_2_ (%), *n*	14	14	14
pH, mean (SD)	7.35 (0.03)	7.35 (0.03)	0.00 (0.01)
pH, *n*	14	14	14
PaCO_2_ (mmHg), mean (SD)	48 (3.8)	48 (3.2)	-0.9 (3.3)
PaCO_2_ (mmHg), *n*	14	14	14
Lactate (mmol/mL), mean (SD)	0.8 (0.1)	0.8 (0.1)	-0.0 (0.1)
Lactate (mmol/mL), *n*	14	14	14
**P then N**			
TV (mL), mean (SD)	344 (61)	339 (64)	5 (19)
TV (mL), *n*	15	15	15
Ppeak (cmH_2_O), mean (SD)	24 (4.3)	23 (3.6)	-0.8 (1.7)
Ppeak (cmH_2_O), *n*	15	15	15
ETCO_2_ (mmHg), mean (SD)	39 (2.9)	38 (3.4)	-1.3 (1.5)
ETCO_2_ (mmHg), *n*	15	15	15
SpO_2_ (%), mean (SD)	99 (1.0)	99 (1.2)	0.1 (0.5)
SpO_2_ (%), *n*	15	15	15
pH, mean (SD)	7.37 (0.04)	7.38 (0.03)	0.01 (0.02)
pH, *n*	15	15	15
PaCO_2_ (mmHg), mean (SD)	46 (4.5)	43 (4.0)	-2.6 (2.5)
PaCO_2_ (mmHg), *n*	15	15	15
Lactate (mmol/mL), mean (SD)	0.8 (0.3)	0.8 (0.3)	0.0 (0.1)
Lactate (mmol/mL), *n*	15	15	15

Abbreviations: 95% CI, 95% confidence interval; ETCO_2_, end-tidal carbon dioxide tension; N, normal saline infusion; P, phenylephrine infusion; PaCO_2_, arterial partial pressure of carbon dioxide; PaO_2_, arterial partial pressure of oxygen; Ppeak, peak inspiratory pressure; SD, standard deviation; SpO_2_, peripheral oxygen saturation; TV, total volume.

Values expressed as mean (SD).

**Table 7:** Hemodynamic and arterial blood gas data (analysis of treatment effect).

Treatment effect	Within-individual difference: N-P
TV (mL), mean (95% CI)	-6 (-14 to 2)
TV (mL), *n*	29
Paired analysis	*P* = 0.13^a^
Ppeak (cmH_2_O), mean (95% CI)	1.0 (0.4 to 1.6)
Ppeak (cmH_2_O), *n*	29
Paired analysis	*P* = 0.003^a^
ETCO_2_ (mmHg), mean (95% CI)	1.3 (0.6 to 1.9)
ETCO_2_ (mmHg), *n*	29
Paired analysis	*P* < 0.001 *=* 0.0006^a^
SpO_2_ (%), mean (95% CI)	0.0 (-0.2 to 0.2)
SpO_2_ (%), *n*	29
Paired analysis	*P* = 0.97^a^
pH, mean (95% CI)	-0.01 (-0.01 to 0.00)
pH, *n*	29
Paired analysis	*P* = 0.06^a^
PaCO_2_ (mmHg), mean (95% CI)	1.7 (0.6 to 2.8)
PaCO_2_ (mmHg), *n*	29
Paired analysis	*P* = 0.003^a^

Abbreviations: 95% CI, 95% confidence interval; ETCO_2_, end-tidal carbon dioxide tension; PaCO_2_, arterial partial pressure of carbon dioxide; PaO_2_, arterial partial pressure of oxygen; Ppeak, peak inspiratory pressure; SpO_2_, peripheral oxygen saturation.

Values expressed as mean (95% CI).

^a^*P* values were calculated using ANOVA for Cross-over design.

**Table 8:** Hemodynamic and arterial blood gas data (carryover effect and period effect).

	Within-individual difference: N-P
**Carryover effect**	
TV (mL), mean (95% CI)	-13 (-102 to 76)
TV (mL), *n*	29
Paired analysis	*P* = 0.81^a^
Ppeak (cmH_2_O), mean (95% CI)	-0.7 (-5.4 to 4.0)
Ppeak (cmH_2_O), *n*	29
Paired analysis	*P* = 0.80^a^
ETCO_2_ (mmHg), mean (95% CI)	0.5 (-3.5 to 4.3)
ETCO_2_ (mmHg), *n*	29
Paired analysis	*P* = 0.85^a^
SpO_2_ (%), mean (95% CI)	1.0 (-0.6 to 2.5)
SpO_2_ (%), *n*	29
Paired analysis	*P* = 0.29^a^
pH, mean (95% CI)	0.05 (0.01 to 0.08)
pH, *n*	29
Paired analysis	*P* = 0.04^a^
PaCO_2_ (mmHg), mean (95% CI)	-5.6 (-10.2 to -1.0)
PaCO_2_ (mmHg), *n*	29
Paired analysis	*P* = 0.049^a^
Lactate (mmol/mL), mean (95% CI)	-0.0 (-0.3 to 0.2)
Lactate (mmol/mL), *n*	29
Paired analysis	*P* = 0.77^a^
**Period effect**	
TV (mL), mean (95% CI)	-2 (-10 to 6)
TV (mL), *n*	29
Paired analysis	*P* = 0.69^a^
Ppeak (cmH_2_O), mean (95% CI)	0.2 (-0.4 to 0.8)
Ppeak (cmH_2_O), *n*	29
Paired analysis	*P* = 0.49^a^
ETCO_2_ (mmHg), mean (95% CI)	-0.0 (-0.7 to 0.6)
ETCO_2_ (mmHg), *n*	29
Paired analysis	*P* = 0.94^a^
SpO_2_ (%), mean (95% CI)	0.1 (-0.1 to 0.3)
SpO_2_ (%), *n*	29
Paired analysis	*P* = 0.26^a^
pH, mean (95% CI)	0.01 (-0.00 to 0.01)
pH, *n*	29
Paired analysis	*P* = 0.08^a^
PaCO_2_ (mmHg), mean (95% CI)	-0.8 (-1.9 to 0.3)
PaCO_2_ (mmHg), *n*	29
Paired analysis	*P* = 0.14^a^
Lactate (mmol/mL), mean (95% CI)	-0.0 (-0.4 to 0.1)
Lactate (mmol/mL), *n*	29
Paired analysis	*P* = 0.66^a^

Abbreviations: 95% CI, 95% confidence interval; ETCO_2_, end-tidal carbon dioxide tension; PaCO_2_, arterial partial pressure of carbon dioxide; PaO_2_, arterial partial pressure of oxygen; Ppeak, peak inspiratory pressure; SpO_2_, peripheral oxygen saturation.

Values expressed as mean (95% CI).

^a^*P* values were calculated using ANOVA for Cross-over design.

Carryover effects were detected in pH and PaCO_2_. There was no period effect among the other hemodynamic and arterial blood gas parameters. There were some differences in peak inspiratory pressure and ETCO_2_ between the two timepoints. No patient had any surgical or other perioperative complications.

## Discussion

Phenylephrine infusion at a rate of 15 μg/min did not have clinically meaningful effects on the PaO_2_ during OLV. However, phenylephrine infusion increased the MAP by about 30%, and decreased the HR and PPV. Phenylephrine infusion did not significantly change the PI and ΔT compared with saline infusion. To the best of our knowledge, the present study is the first to evaluate the clinical effects of phenylephrine infusion on oxygenation and other hemodynamic indices during OLV.

We designed the present study as a crossover trial. The crossover design can decrease the required sample size, as each subject serves as his or her own control. The short-lasting effects of phenylephrine make it suitable for use in a crossover trial. Phenylephrine is a short-acting drug that decreases blood pressure by 50% in 2–3 minutes[10]. We chose a washout time of 10 minutes, as 10–15 minutes is five times the half-life of phenylephrine. We confirmed that there was no carryover effect on the primary or secondary outcomes. As HPV is a biphasic response, it is possible that the order of drug infusion might have affected the PaO_2_. The first phase of HPV begins within 1 minute and reaches a plateau at 15 minutes, while the second phase of HPV begins after 40 minutes of hypoxia and peaks at 2 hours[3]. We considered that the effect of the second phase of HPV was non-significant because the second phase of HPV is reportedly weak[3]. However, the period had a significant effect on the PaO_2_. We usually ventilate patients with an FIO_2_ of around 0.5 during OLV. We do not consider a difference in PaO_2_ of less than 10 mmHg to be clinically meaningful during positive ventilation in a patient with an FIO_2_ of 0.5. We determined the clinically significant difference in PaO_2_ as 30 mmHg during positive ventilation with an FIO_2_ of 1.0, which means a difference of 15 mmHg with an FIO_2_ of 0.5. We believe that using a difference of 30 mmHg in PaO_2_ is appropriate, as previous studies have used a difference of 40 mmHg[8, 11].

The present results show that phenylephrine infusion did not enhance HPV during OLV. This differs from the results of Doering et al.[6], who found that phenylephrine infusion (50–200 μg/min titrated to a 20% increase in MAP) improved the PaO_2_ from 94 ± 8 to 109 ± 12 mmHg in patients with acute respiratory distress syndrome[6]; phenylephrine infusion improved oxygenation in six out of 12 patients[6]. Doering et al.[6] speculated that regional pulmonary vasoconstriction is necessary to improve oxygenation during phenylephrine infusion, as phenylephrine increased mean pulmonary pressure in phenylephrine responders but not in phenylephrine non-responders. One possible explanation for the difference between our results and those of Doering et al.[6] is that the pulmonary arteries in the non-dependent lung in our study were already maximally constricted, so the phenylephrine was not able to constrict these arteries. Tanaka and Dohi[12] showed that phenylephrine bolus injection did not affect the PaO_2_ during OLV. Schloss et al.[4] reported that phenylephrine infusion increased the PaO_2_ in a 12-year-old female who underwent OLV for anterior-posterior supine fusion. Her SpO_2_ was only 90–92% with OLV applying continuous positive airway pressure to the non-dependent lung[4]; however, a phenylephrine infusion of 0.1–0.3 μg/kg/min increased the SpO_2_ to 96–98%[4]. Similarly, Sato and Kato[5] reported that a phenylephrine infusion of 0.1–0.2 μg/kg/min increased the PaO_2_ from 100 mmHg to 150 mmHg in a 59-year-old female who underwent general anesthesia with two-lung ventilation. It seems that the pulmonary vessels in these two previous patients still had the ability to constrict in response to phenylephrine, as there was oxygen in the lungs. It is plausible that phenylephrine infusion may be able to improve oxygenation only when the pulmonary arteries are not fully constricted. Phenylephrine infusion might be effective when the patients are hypoxemic under OLV with continuous positive airway pressure applied to the non-dependent lungs. However, further studies are needed to prove this hypothesis.

We selected the infusion rate of 15 μg/min based on a previous study and our own experiences[13]. Allen et al.[13] demonstrated that a phenylephrine infusion of 25 or 50 μg/min is effective as a prophylactic intervention to prevent maternal hypotension after spinal anesthesia for delivery via cesarean section. As general anesthesia causes less hypotension than spinal anesthesia, we decreased the infusion rate to 15 μg/min. The phenylephrine infusion significantly increased the MAP in the present study, indicating that the infusion rate of 15 μg/min was sufficient to induce vasoconstriction.

Phenylephrine infusion decreased the PPV in the present study, which is similar to the results of a previous study[14]. Rebet et al.[14] showed that bolus intravenous injection of phenylephrine significantly increased corrected flow time measured with esophageal Doppler, and decreased PPV in preload-dependent patients (those with PPV ≥ 13) who underwent general anesthesia. PPV and corrected flow time have been used as cardiac preload indices. It has been shown that α_1_ stimulation constricts the splanchnic capacitance vasculature, which is dilated under general anesthesia, and increases venous return and cardiac preload[15]. Although the ability of PPV as a predictor of fluid responsiveness during OLV is debatable[16], at least two studies have shown that PPV can predict fluid responsiveness during OLV[17, 18]. In the present study, there were some differences in peak inspiratory pressure, ETCO_2_, and PaCO_2_ between the two timepoints. However, we consider these differences to be clinically irrelevant. The increase in ETCO_2_ might have resulted from increased cardiac output due to the aforementioned-increase in cardiac preload.

Our study has several limitations. First, we did not measure cardiac output or intrapulmonary shunt fraction, because the ethics committee of Kagoshima University Hospital declined our request of the insertion of esophageal doppler device or transesophageal echocardiography. We were not able to analyze the effects of phenylephrine on cardiac output or intrapulmonary shunt fraction, although these parameters directly affect oxygenation [3]. Second, it is possible that the anesthesiologists were aware of the group allocation, as phenylephrine infusion significantly increased the MAP. Hence, this could have produced a bias. However, we believe that our strict protocol minimized the effects of this potential bias.

## Conclusion

Phenylephrine infusion does not have clinical effects on oxygenation in patients undergoing OLV. Phenylephrine infusion should be used to stabilize patients’ hemodynamic status, not to improve oxygenation during OLV.

## Supporting information

S1 Fig This is the CONSORT checklist. (DOC)

S1 File Trial protocol. Study protocol in English.
(DOCX)

S2 File Trial protocol. Study protocol in Japanese.
(PDF)

## References

[1] Karzai W, Schwarzkopf K. Hypoxemia during one-lung ventilation: prediction, prevention, and treatment. Anesthesiology. 2009;110(6):1402–11. Epub 2009/05/07. doi: 10.1097/ALN.0b013e31819fb15d . 19417615 10.1097/ALN.0b013e31819fb15d

[2] Brassard CL, Lohser J, Donati F, Bussieres JS. Step-by-step clinical management of one-lung ventilation: continuing professional development. Can J Anaesth. 2014;61(12):1103–21. Epub 2014/11/13. doi: 10.1007/s12630-014-0246-2 . 25389025 10.1007/s12630-014-0246-2

[3] Lumb AB, Slinger P. Hypoxic pulmonary vasoconstriction: physiology and anesthetic implications. Anesthesiology. 2015;122(4):932–46. Epub 2015/01/15. doi: 10.1097/ALN.0000000000000569 . 25587641 10.1097/ALN.0000000000000569

[4] Schloss B, Martin D, Beebe A, Klamar J, Tobias JD. Phenylephrine to Treat Hypoxemia during One-Lung Ventilation in a Pediatric Patient. Thorac Cardiovasc Surg Rep. 2013;2(1):16–8. Epub 2014/11/02. doi: 10.1055/s-0033-1343734 ; PubMed Central PMCID: PMCPMC4176063. 25360404 10.1055/s-0033-1343734 PMC4176063

[5] Sato K, Kato M. [Improvement in oxygenation by alpha stimulant]. Masui. 2001;50(10):1118–20. Epub 2001/11/20. . 11712347

[6] Doering EB, Hanson CW 3rd, Reily DJ, Marshall C, Marshall BE. Improvement in oxygenation by phenylephrine and nitric oxide in patients with adult respiratory distress syndrome. Anesthesiology. 1997;87(1):18–25. Epub 1997/07/01. . 9232130 10.1097/00000542-199707000-00004

[7] Biais M, Ouattara A, Janvier G, Sztark F. Case scenario: respiratory variations in arterial pressure for guiding fluid management in mechanically ventilated patients. Anesthesiology. 2012;116(6):1354–61. Epub 2012/04/26. doi: 10.1097/ALN.0b013e318256ee28 . 22531335 10.1097/ALN.0b013e318256ee28

[8] Montes FR, Pardo DF, Charris H, Tellez LJ, Garzon JC, Osorio C. Comparison of two protective lung ventilatory regimes on oxygenation during one-lung ventilation: a randomized controlled trial. J Cardiothorac Surg. 2010;5:99 Epub 2010/11/04. doi: 10.1186/1749-8090-5-99 ; PubMed Central PMCID: PMCPMC2987929. 21044330 10.1186/1749-8090-5-99 PMC2987929

[9] Li T, Yu T, Hawkins BS, Dickersin K. Design, Analysis, and Reporting of Crossover Trials for Inclusion in a Meta-Analysis. PLoS One. 2015;10(8):e0133023 Epub 2015/08/19. doi: 10.1371/journal.pone.0133023 ; PubMed Central PMCID: PMCPMC4540315. 26284684 10.1371/journal.pone.0133023 PMC4540315

[10] Xia J, Sun Y, Yuan J, Lu X, Peng Z, Yin N. Hemodynamic effects of ephedrine and phenylephrine bolus injection in patients in the prone position under general anesthesia for lumbar spinal surgery. Exp Ther Med. 2016;12(2):1141–6. Epub 2016/07/23. doi: 10.3892/etm.2016.3432 ; PubMed Central PMCID: PMCPMC4950852. 27446334 10.3892/etm.2016.3432 PMC4950852

[11] Unzueta MC, Casas JI, Moral MV. Pressure-controlled versus volume-controlled ventilation during one-lung ventilation for thoracic surgery. Anesth Analg. 2007;104(5):1029–33, tables of contents. Epub 2007/04/26. doi: 10.1213/01.ane.0000260313.63893.2f . 17456648 10.1213/01.ane.0000260313.63893.2f

[12] Tanaka M, Dohi S. [The effects of ephedrine and phenylephrine on arterial partial pressure of oxygen during one-lung ventilation]. Masui. 1994;43(8):1124–9. Epub 1994/08/01. . 7933491

[13] Allen TK, George RB, White WD, Muir HA, Habib AS. A double-blind, placebo-controlled trial of four fixed rate infusion regimens of phenylephrine for hemodynamic support during spinal anesthesia for cesarean delivery. Anesth Analg. 2010;111(5):1221–9. Epub 2010/05/25. doi: 10.1213/ANE.0b013e3181e1db21 . 20495139 10.1213/ANE.0b013e3181e1db21

[14] Rebet O, Andremont O, Gerard JL, Fellahi JL, Hanouz JL, Fischer MO. Preload dependency determines the effects of phenylephrine on cardiac output in anaesthetised patients: A prospective observational study. Eur J Anaesthesiol. 2016;33(9):638–44. Epub 2016/05/11. doi: 10.1097/EJA.0000000000000470 . 27164015 10.1097/EJA.0000000000000470

[15] Appleton C, Olajos M, Morkin E, Goldman S. Alpha-1 adrenergic control of the venous circulation in intact dogs. J Pharmacol Exp Ther. 1985;233(3):729–34. Epub 1985/06/01. . 2861278

[16] Raphael J, Regali LA, Thiele RH. Hemodynamic monitoring in thoracic surgical patients. Curr Opin Anaesthesiol. 2017;30(1):7–16. Epub 2016/12/29. doi: 10.1097/ACO.0000000000000408 . 28030449 10.1097/ACO.0000000000000408

[17] Lee JH, Jeon Y, Bahk JH, Gil NS, Hong DM, Kim JH, et al Pulse pressure variation as a predictor of fluid responsiveness during one-lung ventilation for lung surgery using thoracotomy: randomised controlled study. Eur J Anaesthesiol. 2011;28(1):39–44. Epub 2010/11/23. doi: 10.1097/EJA.0b013e32834089cf . 21088596 10.1097/EJA.0b013e32834089cf

[18] Fu Q, Duan M, Zhao F, Mi W. Evaluation of stroke volume variation and pulse pressure variation as predictors of fluid responsiveness in patients undergoing protective one-lung ventilation. Drug Discov Ther. 2015;9(4):296–302. Epub 2015/09/16. doi: 10.5582/ddt.2015.01046 . 26370528 10.5582/ddt.2015.01046

