# Supplementary material for: Phenylephrine does not improve oxygenation during one-lung ventilation: A randomized, double-blind, cross-over study
Source: PLoS One. 2018 Apr 9;13(4):e0195576. doi: 10.1371/journal.pone.0195576 (PMC5891027; doi:10.1371/journal.pone.0195576)
Supplement: S1 File — Study protocol in English. (DOCX) [file pone.0195576.s002.docx]

**the clinical trial protocol**

**Title:** The investigation of the effects of phenylephrine on oxygenation during one-lung ventilation; a randomized, double-blind, cross-over study

**Review Board:** Approval Status: Approved

**Approval Number:** 27-137

**Approval Date:** 11/11/2015

**Board Name:** the ethics committee of Kagoshima University Hospital

No.1

Application #27-137

Application Date 9/25/2015

**Clinical trial application form for the ethics committee**

Date 9/17/2015

Dear Chair of Kagoshima University Hospital

Applicant: Dept. of Operating Room

Fellow Kohei Godai

Dept. of Operating Room

Associate Professor Akira Matsunaga

We here apply for the review of the following clinical trial.

**1. Study title:** The investigation of the effects of phenylephrine on oxygenation during one-lung ventilation; a randomized, double-blind, cross-over study

**2. Sponsor:** Dept. of Operating Room, Associate Professor Akira Matsunaga

**3. Collaborators:** Dept. of Operating Room, Fellow Kohei Godai

Dept. of Anesthesiology, Associate Professor Maiko Hasegawa

Dept. of Anesthesiology, Professor Yuichi Kanmura

**4. Narrative objectives:** To evaluate the effects of phenylephrine infusion on oxygenation during one-lung ventilation in patients undergoing thoracic surgery

Basic protocol: This is a prospective, randomized, double-blind, cross-over study. Patients are randomly allocated to two groups. The N-P group initially has OLV maintained with normal saline infusion (20 mL/h) for 30 minutes; after a 10 minute interval, OLV is then maintained with phenylephrine infusion (15 μg/min) for 30 minutes. The P-N group has the drug-infusion in the reverse order.

The primary outcome: arterial partial pressure of oxygen.

The Secondary outcomes: mean arterial pressure, heart rate, pulse pressure variation, perfusion index, and difference between bladder and skin temperature.

**5. Possible adverse effects**

We use phenylephrine during the everyday practice. The dose and administration route adhere the manufacturer’s recommendations. Data will be anonymized.

Phenylephrine may cause hypertension or bradycardia. We will treat hypertension with nicardipine or diltiazem. We will use atropine for bradycardia.

**6. The place of investigation**

Kagoshima University Hospital, Operating Rooms

**7. People in charge of managing collected data**

Assistant administrator: Dept. of Operating Room, Associate Professor Akira Matsunaga

Administrator: Dept. of Anesthesiology, Associate Professor Maiko Hasegawa

**8. The study title in English**

The effects of phenylephrine on oxygenation during one-lung ventilation; a randomized, double-blind, cross-over study

No. 1-①

**Study protocol**

**Title:** The investigation of the effects of phenylephrine on oxygenation during one-lung ventilation; a randomized, double-blind, cross-over study

**1. The research members**

①Collaboration institute: None

②Sponsor: Dept. of Operating Room, Associate Professor Akira Matsunaga

Collaborators: Dept. of Operating Room, Fellow Kohei Godai

Dept. of Anesthesiology, Associate Professor Maiko Hasegawa

Dept. of Anesthesiology, Professor Yuichi Kanmura

③Consignment: None

**2. Invasiveness and insurance**

①Invasiveness: +

②Intervention: +

③Insurance: +

**3. Clinical trial registration**

Registration: +

UMIN Clinical Trials Registry

**4. Inclusion criteria and sample size**

①Adult patients with ASA physical status 1-3 undergoing elective thoracic surgery in the lateral decubitus position with at least 70 minutes of one-lung ventilation

②Sample size: 40

**5. Scientific background**

①Hypoxemia during one-lung ventilation (OLV) occurs in 5–10% of patients, and may affect the patient safety. HPV is attenuated by intravenous vasodilators such as prostacyclin or calcium antagonists, and thus these drugs worsen the oxygenation status.

②The aim of the present study is to evaluate the clinical effects of phenylephrine infusion on oxygenation during OLV in patients undergoing thoracic surgery.

③Phenylephrine is an α1 adrenergic receptor agonist that causes pulmonary vasoconstriction. Phenylephrine administration may effectively enhance HPV; however, there is little evidence that phenylephrine augments HPV in clinical situations. Two case reports showed that phenylephrine infusion improved oxygenation during general anesthesia.

④Methods

Before induction of general anesthesia, a thoracic epidural catheter (17G Tuohy needle, Hakko disposable epidural catheter; Hakko, Japan) is placed at T4 to T7 according to the incision level, and 3 mL mepivacaine 1% without epinephrine is administered. General anesthesia is induced with target-controlled infusion of propofol (2–4 μg/mL), remifentanil (0.3 μg/kg/min), and rocuronium (0.6 mg/kg). Anesthesia is maintained with target-controlled infusion of propofol (2–4 μg/mL) and remifentanil (0.1–0.5 μg/kg/min), and intermittent bolus administration of rocuronium (10 mg). Bispectral index (BIS) values are monitored using a BIS Quatro sensor (Covidien, Mansfield, MA, USA), and the propofol infusion rate is adjusted to maintain BIS values between 40 and 60. No volatile anesthetics are used. The rate of remifentanil infusion is tailored to control hemodynamic responses. Rocuronium is used to maintain a train-of-four ratio of 1 or less (TOF-Watch SX, Organon, Ireland). Heart rate (HR), direct arterial blood pressure, electrocardiogram, BIS, peripheral oxygen saturation (SpO2), end-tidal carbon dioxide tension (ETCO2), PPV, perfusion index (PI) derived from the pulse oximeter plethysmographic waveform, bladder temperature, and skin temperature of the hand are continuously monitored (Life Scope J, Nihon Kohden, Japan). Intravenous fluid therapy consists of 6% hydroxyethyl starch in saline (Voluven, Fresenius Kabi Japan, Japan) at a rate of 80 ml/h, and 4.3% dextrose solution (Soldem 3A, Terumo, Japan) at a rate of 20 ml/h. If the mean arterial pressure (MAP) is less than 50 mmHg, a bolus of ephedrine (4 mg) was administered.

The trachea is intubated with a left-sided double-lumen tube (DLT) (Blue Line, Smiths Medical International, UK: 35 or 37 F for males and 32 or 35 F for females). Positioning of the DLT is confirmed by fiberoptic bronchoscopy before and after placing the patient in the lateral decubitus position. Lung separation was confirmed by auscultation. During OLV, the lumen of the non-dependent lung is left open to air. The patients’ lungs are ventilated using an Aisys Pro anesthetic machine (GE Healthcare, Chicago, IL, USA) with an inspired oxygen fraction of 1.0, tidal volume of 5–7 mL/kg of ideal bodyweight, and respiratory rate of 12 breaths/min; this is adjusted to maintain an ETCO2 of 35–45 mm Hg and a positive end-expiratory pressure of 6 cmH2O. The patients are randomly allocated to one of two groups using internet-based software (Research Randomizer version 4.0, retrieved on October 13, 2016 from http://www.randomizer.org/). The allocation is blinded for the patients, anesthesiologists, and surgeons. In the N-P group, 10 minutes after each patient’s chest is opened, OLV is initially maintained with a normal saline infusion (20 mL/h) for 30 minutes; after a 10 minute interval, OLV is then maintained with a phenylephrine infusion (15 μg/min) for 30 minutes. In the P-N group, 10 minutes after each patient’s chest is opened, OLV is initially maintained with a phenylephrine infusion (15 μg/min) for 30 minutes; after a 10 minute interval, OLV is then maintained with a normal saline infusion (20 mL/h) for 30 minutes. At the end of each drug infusion, arterial blood analysis is performed with a blood gas analyzer (ABL700, Radiometer, Denmark), and the MAP, HR, PPV, and PI were recorded. These measurements are recorded before any major pulmonary vessel clipping. The anesthetic management after these measurements is at the discretion of the anesthesia care provider.

**Inclusion criteria**

Adult patients with ASA physical status 1-3 undergoing elective thoracic surgery in the lateral decubitus position with at least 70 minutes of one-lung ventilation

**Exclusion criteria**

History of stroke, uncompensated cardiac disease, or bradyarrhythmias.

**Drugs**

Phenylephrine, Normal Saline, Nicardipine, Diltiazem, Atropine

⑤Duration of the study

After approval-12/31/2017

**6. Possible effects and side effects**

①Possible effects

This study improves the safety of perioperative OLV.

②Possible side effects

Hypertension, bradycardia, the other side effects.

③Possible risks

The risk of developing hypertension is low.

④Management of severe injury

We appropriately manage the injury if severe injury occurs. We will report the incidence of severe injury to the chair of Kagoshima University Hospital.

⑤Necessary measures to compensate the subjects for any injury occurring in the study

Any injury caused by participating in the study is covered by the insurance.

**7. Management of the patients after completion of the intervention**

The anesthetic management after these measurements was at the discretion of the anesthesia care provider.

**8. The other treatment**

None

**9. There is no risk to find important genetic information in this study.**

**10. Management of the collected data**

①Storage of the collected data

We anonymize the information and store the data in the locked chest.

②Destruction of documents

We will destroy documents 5 years after publication of the results.

③Potential usage of collected data in future study

+

④Potential usage of collected data in the other institutions

None

**11. Funding information**

①Funding information.

None

②conflict of interest

None

**12. Compensation for cooperating in a clinical study**

None

**13. Monitoring and audit**

Monitoring

①Monitor: Dept. of Operating Room, Assistant Professor Tamotsu Kuniyoshi

①Monitoring protocol: Monitoring will be done twice (during the study, and after completion of the study).

Audit

①Dept. of Anesthesiology, Fellow Tomotsugu Yamada

②Audit will be done appropriate time during the study.

**14. Informed consent**

After explanation of the study to subjects, their written informed consent is obtained in order to conduct the study.

**15. Protection of privacy**

Personal information, such as names, is removed from the data and samples of subjects, and the data and samples are anonymized by assigning new codes or numbers instead. The correspondence table matching the subjects and these codes [numbers] is strictly protected to prevent it from being leaked). The data is strictly stored in a locked place.

**16. Contact form**

Researchers are available for contact from participants.

**17. Publication of study outcomes**

①When protocol changes.

②Around November annually.

③After completion of the study.

④etc.

**Contact Information**

Associate Professor Akira Matsunaga

Department of Operating Room

Kagoshima University Hospital

8-35-1 Sakuragaoka, Kagoshima 890-8520, Japan

Phone: +81-99-275-5430, Fax: +81-99-265-1642
