# Supplementary material for: Phenylephrine does not improve oxygenation during one-lung ventilation: A randomized, double-blind, cross-over study
Source: PLoS One. 2018 Apr 9;13(4):e0195576. doi: 10.1371/journal.pone.0195576 (PMC5891027; doi:10.1371/journal.pone.0195576)
Supplement: S2 File — Study protocol in Japanese. (PDF) [file pone.0195576.s003.pdf]

承認番号	27－137

臨床研究倫理審査結果通知書

平成 27 年 11 月 11 日

手術部

五　代　幸　平　殿

鹿児島大学医学部・歯学部附属病院長

熊　本　一　朗

臨床研究課題名

片側肺換気下での血管収縮薬フェニレフリンによる酸素化改善効果の検討（ランダム化・二重盲検・交差試験）

研究責任者　　手術部　　松　永　　明

上記課題に係る実施計画書等を、平成２７年１１月９日開催の鹿児島大学医学部・歯学部附属病院臨床研究倫理委員会で審査し、下記のとおり判定されましたので、通知します。

記

判定	承認
条件（理由）	

➢ 委員会名　　鹿児島大学医学部・歯学部附属病院　臨床研究倫理委員会

➢ 所在地　　〒890-8520　鹿児島県鹿児島市桜ヶ丘八丁目 35 番地 1 号

➢ 審査委員（8 名）

本研究を審査した委員を以下のとおり示す。

☑ 委員長　井戸　章雄　（鹿児島大学医学部・歯学部附属病院　消化器内科　教授）
☐ 副委員長　野口　和行　（鹿児島大学医学部・歯学部附属病院　歯周病科　教授）
☐ 審査員　上野　真一　（鹿児島大学大学院医歯学総合研究科　臨床腫瘍学講座　教授）
☑ 審査員　橋口　照人　（鹿児島大学医学部・歯学部附属病院　検査部　教授）
☑ 審査員　堀内　正久　（鹿児島大学大学院医歯学総合研究科　衛生学・健康増進医学　教授）
☑ 審査員　幾留　秀一　（鹿児島女子短期大学　学長）
☑ 審査員　石窪　奈穂美（消費生活アドバイザー）
☑ 審査員　平手　賢治　（志學館大学　法学部　准教授）

➢ 審査した書類

☑ 申請書
☑ 実施計画書
☑ 説明文書
☑ 同意書
☐ ホームページ用公開文書
☐ その他（　　　　　）

➢ 審議事項

☑ 研究の実施の可否
☐ 研究の継続の適否
　☐ 研究に関する変更
　☐ 重篤な有害事象等
　☐ 安全性情報等
☐ その他（　　　　）

➢ 審査区分

☑ 委員会審査（審査日：平成 27 年 11 月 9 日）（☑ 面接、☐ 書類）
☐ 迅速審査　（審査日：平成　年　月　日）（☐ 面接、☐ 書類）

記

本委員会は、「人を対象とする医学系研究に関する倫理指針」（平成 26 年文部科学省・厚生労働省告示第 3 号）に基づき、独立かつ公正な立場で科学的合理性のもとに、医学的かつ倫理的妥当性について審議を行う委員会であることを宣言する。

鹿児島大学医学部・歯学部附属病院
臨床研究倫理委員会
委員長　井戸　章雄

（第１号様式）

★	受付番号	27－137
★	受理年月日	H27．9．25

臨床研究倫理審査申請書

平成２７年９月１７日

鹿児島大学医学部・歯学部附属病院長　殿

【申請者】
所　　属　手術部
職・氏名　　医員　五代　幸平

【部門科長】　手術部
職・氏名　准教授　松永　明

下記について、審査を申請します。

記

１．研究課題名	片側肺換気下での血管収縮薬フェニレフリンによる酸素化改善効果の検討（ランダム化・二重盲検・交差試験）
２．研究責任者	手術部　准教授　松永　明
３．分担者所属氏名	手術部　医員　五代　幸平<br>麻酔科　准教授　長谷川　麻衣子<br>麻酔科　教授　上村　裕一

４．研究の目的及び実施計画の概要及び評価項目
本研究は鹿児島大学病院単独で行う研究である。

目的：片側肺換気においては低酸素血症が 5-10%の症例で生じると報告されている。片側肺換気下において血管収縮薬フェニレフリンによる酸素化改善効果の有無を検討することを目的とする。

概要：予定肺手術患者において片側肺換気下でフェニレフリンおよび生理食塩水の酸素化改善効果を以下の２群に分け、ランダム化・二重盲検・交差試験で検討する。フェニレフリン先行投与群・生食先行投与群。

主要評価項目：動脈血酸素分圧

副次評価項目：副作用（高血圧・徐脈）の頻度

5．研究の実施により生じる倫理上の問題点及び副作用とその対処法等

予想される倫理上の問題：フェニレフリンは通常の麻酔中に使用される薬剤であり、使用量・方法共に保険診療の範囲内である。採血量も 3.0ml であり、通常行われる手術中の採血と同等量である。対象者の選定、説明書と同意については鹿児島大学医学部・歯学部附属病院臨床研究倫理委員会の審査を経て、その承諾事項を遵守して実行する。データは連結可能匿名化し対応表を作成し、個人を特定できないように配慮する。またすべての個人情報と対応表は、それぞれ麻酔科研究室内の鍵のかかる保管庫に保存し厳重に管理する。研究終了について報告した日から 5 年を経過した日、又は研究結果の最終公表について報告した日から 3 年を経過した日のいずれか遅い日までの期間を経過した後はシュレッダー等で粉砕するなど、個人情報がわからないようにした上で破棄する。松永・上村の監督の下、本研究の割り付けは長谷川が行い、情報収集および解析は五代が行う。

副作用とその対処法：フェニレフリンは主に昇圧薬として使用されており，副作用として高血圧・徐脈が生じ得る。高血圧に対しては、降圧薬であるニカルジピンもしくはジルチアゼムを投与し血圧を正常化する。徐脈に対しては硫酸アトロピンを投与し、徐脈を解消する。

6．研究の実施場所

鹿児島大学医学部・歯学部附属病院、手術室

7．当該研究に係る個人情報の管理者等

副保護管理者：　手術部　准教授　松永　明
保護担当者　：　麻酔科　准教授　長谷川　麻衣子

8．研究課題名（英文表記）

The effects of phenylephrine on oxygenation during one-lung ventilation; a randomized, double-blind, cross-over study

（第１－①様式）

臨 床 研 究 実 施 計 画 書

研究課題名：	片側肺換気下での血管収縮薬フェニレフリンによる酸素化改善効果の検討（ランダム化・二重盲検・交差試験）

１．研究の実施体制

①共同研究機関の名称

なし

②研究者等の氏名

【研究責任者】

鹿児島大学病院

		講習受講歴
手術部		
准教授	松永　明	☑有

【研究分担者】

鹿児島大学病院

手術部		
医員	五代　幸平	☑有
麻酔科		
准教授	長谷川　麻衣子	☑有
教授	上村　裕一	☑有

③研究に関する業務の委託：無

２．侵襲性と介入及び臨床保険の有無について

①侵襲性：有

②介入：有

③臨床保険の加入：加入手続き中

３．臨床研究に関する情報公開の方法

・データベースへの登録：有

☑国立大学附属病院長会議（UMIN）

４．被験者の選定の方針及び症例数

①被験者の選定方針：

鹿児島大学病院にて予定肺手術のため、片側肺換気および動脈血液ガス分析を必要とし全身麻酔を受ける患者。

②症例数：40 症例

５．研究の社会的、学術的な意義、目的、科学的合理性の根拠、方法及び期間

①意義：肺手術においては片側肺換気が必要となるが、片側肺換気においては低酸素血症が 5-10%の症例で生じると報告されている。低酸素血症

では組織での酸素不足により、臓器障害を来す。低酸素血症を改善することが出来れば、片側肺換気の安全性を高めることが可能であり、学術的だけでなく社会的にも意義が大きいと考えられる。今回の方法で 30mmHg の動脈血酸素分圧の改善があれば、臨床的に有意義と考えられる。

②目的：片側肺換気下において血管収縮薬フェニレフリンによる酸素化改善効果の有無を検討することを目的とする。

③科学的合理性の根拠：片側肺換気中には低酸素性肺血管収縮という生理学的機構が働き、非換気肺への血流を減少させる。ヒトでの研究で血管収縮薬であるフェニレフリンは肺血管も収縮させるため、この低酸素性肺血管収縮を増強し、酸素化を改善すると考えられている（Anesthesiology 1997; 87: 18-25.）。しかし、フェニレフリンの酸素化改善効果については臨床的に十分検討されていない。そのため、本研究でその効果を検討することは科学的に妥当だと判断する。承認薬剤であり通常麻酔中使用されているため、安全性は担保されている。

④方法：同意取得後の患者に対し乱数表を用いたインターネット上のプログラム Research Randomizer Version 4.0 (http://www.randomizer.org) を用いて、フェニレフリン・生食群および生食・フェニレフリン群の 2 群にランダムに群分けする。群分けについては患者および麻酔担当者に対して盲検化する。松永・上村の監督の下、本研究の割り付けは長谷川が行い、情報収集および解析は五代が行う。

全身麻酔導入および動脈血液採取用のカテーテルを挿入する。その後、片側肺換気を開始する。換気状態が一定となったことを確認したのち、以下の介入を行う。

1 群の患者には片側肺換気中に、先ずフェニレフリン（15μg/min）を 30 分間持続静注したのち動脈血液ガス分析のため動脈採血（1.5ml）を行う。10 分間の回復期間ののち、次に生理食塩水（20ml/h）を 30 分間持続静注し、再度動脈血液ガス分析のため動脈採血（1.5ml）を行う。

もう 1 群の患者では、先ず生理食塩水（20ml/h）を 30 分間持続静注したのち動脈血液ガス分析のため動脈採血（1.5ml）を行う。10 分間の回復期間ののち、次にフェニレフリン（15μg/min）を 30 分間持続静注し、再度動脈血液ガス分析のため動脈採血（1.5ml）を行う。

このフェニレフリンおよび生理食塩水の投与量は通常投与量の範囲である。この介入で動脈採血（1.5ml）を 70 分間で 2 回行うこととなるが、これも通常の肺手術で行う採血と同等である。その他、動脈血液採取用カテーテル挿入、片側肺換気をはじめとする麻酔の手技も通常診療と同一手技である。

介入終了後は麻酔担当者が適切な麻酔を継続する。

介入中に高血圧（平均血圧 110mmHg 以上）を生じた場合は、降圧薬であるニカルジピン（0.4mg）もしくはジルチアゼム（5mg）を投与し血圧を正常化する。徐脈（心拍数 40 回/分以下）を生じた場合には、硫酸ア

トロピン（0.5mg）を投与し徐脈を解消する。高血圧および徐脈が生じた場合は、その頻度を記録する。

【選定基準】
1）予定肺手術のため、片側肺換気および動脈血液ガス分析を必要とし全身麻酔を受ける患者。
2）20歳以上75歳以下の患者

【除外基準】
1）心筋梗塞および心不全の既往
2）脳梗塞および脳出血の既往
3）徐脈性不整脈の患者

【使用する薬剤・医療機器について】
・フェニレフリン（ネオシネジン・コーワ注）、興和株式会社
・生理食塩水（生理食塩液 PL「フソー」）、扶桑薬品工業株式会社
・ニカルジピン（ペルジピン注射液）アステラス製薬株式会社
・ジルチアゼム（ヘルベッサー注射用）田辺三菱製薬株式会社
・アトロピン（アトロピン硫酸塩注 0.5mg）田辺三菱製薬株式会社

⑤総研究期間：臨床研究承認日 ～ 平成29年12月31日
登録期間：臨床研究承認日 ～平成29年12月31日

6．予想される効果と副作用又は研究対象者に及ぼす不利益及びそれに対する対応とこれらの総合的評価
①効果：本研究に参加した場合、フェニレフリンの低酸素性肺血管収縮を増強効果により酸素化が改善する可能性がある。また、将来の片側肺換気が必要な患者の安全性向上に貢献することができる。

②副作用又は研究対象者に及ぼす負担及びリスクとその対処法等：
高血圧（発生率不明）：降圧薬であるニカルジピンもしくはジルチアゼムを投与し血圧を正常化する。
徐脈（発生率不明）：硫酸アトロピンを投与し徐脈を解消する。発生率は不明だが、添付文書では「アトロピンにより容易に回復する。」と記載されている。
その他の副作用：添付文書によると総症例384例中副作用が報告されたのは26例（6.77%）で、主な症状は頭痛12件（3.13%）、手足のしびれ感・ふるえ感5件（1.30%）等であった。しかし、全身麻酔中においては患者の意識及び痛みは消失しているため、頭痛や手足のしびれ感・ふるえ感は生じないと考える。

③予測されるリスク及び利益の総合的評価：
フェニレフリンの投与量は通常投与量の範囲であり、重篤な高血圧や徐脈が発生するリスクは低いと考えられる。片側肺換気中の安全性を高めるという意義を考慮すると、総合的に利益が大きいと考える。

④重篤な有害事象が発生した際の対応：
重篤な有害事象が発生した場合は速やかに対処する。症状が未知の副作用や重篤と判断される場合は、鹿児島大学病院の"重篤な有害事象発生時の手順書"に従い、早急に研究代表者、鹿児島大学病院長へ報告する。

⑤研究によって生じた健康被害に対する補償について
研究に関する臨床保険に加入する。
健康被害が発生した場合は、保険診療で対処する。本研究で使用する試験薬品は、既に対象疾患に対する適応を取得しており、いずれも市販されているため、本研究に定める用法、用量の指示に従い服用した副作用に起因する健康被害は、「医薬品副作用被害救済制度」による救済給付申請の対象になる。

7．研究対象者への研究実施後における医療の提供に対する対応
本研究は手術中の麻酔薬の投与方法を比較検討する研究であり、研究終了後の治療方法に直接的な影響をおよぼす研究ではない。介入終了後は麻酔担当者が適切な麻酔を継続する。

8．その他の治療法
当該研究に参加しない場合、麻酔担当者が適切な全身麻酔およびその他の麻酔を提供する。

9．本研究では、研究対象者の健康・子孫に受け継がれ得る遺伝的特徴に関する重要な知見が得られる可能性はない。

10．得られた試料・情報について
①試料・情報の保管の方法：
データは連結可能匿名化し対応表を作成し、個人を特定できないように配慮する。またすべての個人情報と対応表は、それぞれ麻酔科研究室内の別の鍵のかかる保管庫に保存し厳重に管理する。

②廃棄の方法：
研究終了について報告した日から5年を経過した日、又は研究結果の最終公表について報告した日から3年を経過した日のいずれか遅い日までの期間を経過した後はシュレッダー等で粉砕するなど、個人情報がわからないようにした上で破棄する。

③研究対象者等から同意を受ける時点では特定されない将来の研究のために用いられる可能性：有
本研究のデータを他の研究に使用する場合は、鹿児島大学病院臨床研究倫理委員会に研究計画書を提出し承認された研究のみに使用する。

④他の研究機関に提供する可能性と想定される内容：無

11. 資金源等、関係機関との関係及び利益相反について

①資金源について：

データ解析に関する費用は鹿児島大学病院麻酔科の研究費（医歯学総合研究科教育研究助成金）で実施する。その他の診療・検査にかかる費用は、通常の保険診療の範囲で患者が負担する。

②関係機関との関係及び利益相反：データ解析に関する費用は鹿児島大学病院麻酔科の研究費（医歯学総合研究科教育研究助成金）で実施するため、開示すべき利益相反はない

12. 研究に参加することについて研究協力費の有無：無

13. モニタリング及び監査の実施体制及び実施手順

【モニタリング】

①実施体制：手術部　助教　國吉　保

①実施手順：モニターは、臨床研究責任医師等に対して臨床研究開始前・実施中及び終了後に 1 回ずつモニタリングを実施し、次の事項を確認する。1）被験者の人権保護、 安全持及び福祉向上が図られていること、具縦域には個人情報やデータの管理に問題がないか、2）臨床研究が最新の臨床研究実施計画書を遵守していること、3）臨床研究責任医師から報告されたデータ等が正確かつ完全で原資料等の関連記録に照らして検証できること。

【監査】

①実施体制：麻酔科　医員　山田　知嗣

②実施手順：臨床研究実施中又は臨床研究の終了後の適切な時期に監査を実施する。監査担当者は、以下の内容を確認しながら臨床研究実施計画書ごとに監査計画書を作成し、自ら臨床研究を実施する者に提出し了承を得る。監査計画書には次の事項を記載する。1）監査対象の研究に係る事項(臨床研究課題名、臨床研究実施計画書番号等)、2）監査スケジュール、3）監査範囲（監査対象項目、監査対象資料を含む）、4）臨床研究責任医師への報告方法（報告・連絡窓口等を含む）、5）監査関連資料の流れ（監査計画書、監査報告書等の提出先等）、6）監査実施期間（被監査対象を確認する期間）、7）その他必要と考えられる事項

14. インフォームド・コンセントについて（説明文書・同意書）

別紙のとおり説明文書に基づき、対象患者に対し十分な説明を行い、十分な理解が得られたうえで、研究の参加について原則患者自身に同意書に署名いただく。

代諾者から同意を頂く場合

【代諾者の選定方針】
1．任意後見人，親権者，後見人や保佐人が定まっているときはその人
2．提供者本人の配偶者，成人の子，父母，成人の兄弟姉妹若しくは孫，祖父母，同居の親族又はそれらの近親者に準ずると考えられる人

15. 個人情報の取り扱いについて
データは連結可能匿名化し対応表を作成し、個人を特定できないように配慮する。またすべての個人情報と対応表は、それぞれ麻酔科研究室内の別の鍵のかかる保管庫に保存し厳重に管理する。

16. 研究対象者からの問い合わせ時の対応
研究者が誠意をもって対応する。

17. 研究機関の長への報告内容及び方法
① 実施計画の変更／必要に応じて随時
② 研究の進捗状況報告書について／毎年 11 月頃
③ 研究究終了報告書について／研究終了時
④ その他「人を対象とした医学研究に関する倫理指針」を遵守し、遅滞なく報告する。
